# Phytoplankton Distribution in Relation to Environmental Drivers on the North West European Shelf Sea

**DOI:** 10.1371/journal.pone.0164482

**Published:** 2016-10-13

**Authors:** Beatrix Siemering, Eileen Bresnan, Stuart C. Painter, Chris J. Daniels, Mark Inall, Keith Davidson

**Affiliations:** 1 Scottish Association for Marine Science, Scottish Marine Institute, Oban, United Kingdom; 2 MSS Marine Laboratory, Aberdeen, United Kingdom; 3 National Oceanographic Centre, Southampton, United Kingdom; 4 Department of Geosciences, Grant Institute, University of Edinburgh, Edinburgh, UK; University of Vigo, SPAIN

## Abstract

The edge of the North West European Shelf (NWES) is characterised by a steep continental slope and a northward flowing slope current. These topographic/hydrographic features separate oceanic water and shelf water masses hence potentially separate phytoplankton communities. The slope current may facilitate the advective transport of phytoplankton, with mixing at the shelf edge supporting nutrient supply and therefore phytoplankton production. On the west Scottish shelf in particular, little is known about the phytoplankton communities in and around the shelf break and adjacent waters. Hence, to improve our understanding of environmental drivers of phytoplankton communities, biological and environmental data were collected on seven cross-shelf transects across the Malin and Hebridean Shelves during autumn 2014. Density profiles indicated that shelf break and oceanic stations had a 100 m deep mixed surface layer while stations on the shelf were generally well mixed. Analysis of similarity and multidimensional scaling of phytoplankton counts revealed that phytoplankton communities on the shelf were significantly different to those found at the shelf break and at oceanic stations. Shelf stations were dominated by dinoflagellates, with diatoms contributing a maximum of 37% of cells. Shelf break and oceanic stations were also dinoflagellate dominated but displayed a lower species diversity. Significant difference between shelf and shelf break stations suggested that the continental slope limited cross shelf phytoplankton exchange. Northern and southern phytoplankton communities on the shelf were approximately 15% dissimilar while there was no latitudinal gradient for stations along the slope current, suggesting this current provided south to north connectivity. Fitting environmental data to phytoplankton ordination showed a significant relationship between phytoplankton community dissimilarities and nutrient concentrations and light availability on the shelf compared to shelf break and oceanic stations in the study area.

## Introduction

Phytoplankton account for roughly half of global primary production [1] with an estimated 25% of global production taking place in continental shelf areas, which account for less than 10% area and 0.5% of the total volume of the ocean [2]. Shelf sea phytoplankton are therefore particularly important for global carbon cycling [3] and also form the base of marine food webs underpinning shelf sea fisheries and aquaculture [4]. Coastal blooms of harmful phytoplankton species also pose a threat to fishing and aquaculture industries as well as to tourism and human health [5, 6]. The EU Marine Strategy Framework Directive (MSFD) requires member states to assess if their plankton communities achieve ‘*Good Environmental Status*’ by 2020. Thus there is an increasing requirement to understand the diversity dynamics of phytoplankton communities in all areas of the North West European Shelf (NWES) and how these relate to environmental conditions [7].

Topographic features such as the shelf edge may play a key role in stimulating phytoplankton production through their generation of mixing [8, 9]. In N.W. European waters the 200 m deep continental shelf and adjacent oceanic waters are separated at the shelf break by a steep slope, alongside which the European Slope Current (ESC) is steered. While the ESC and shelf break inhibit direct oceanic-shelf water interactions [10], chlorophyll fluorescence measurements suggest that this boundary between oceanic water and shelf water can provide optimal growth conditions for phytoplankton [11].

Despite its importance, phytoplankton distribution across the NWES shelf seas are relatively poorly studied in comparison to the hydrography of the area [10, 12, 13],particularly the region west of Scotland. The few existing studies conducted in this location have generally found different phytoplankton communities on the shelf compared to adjacent oceanic waters [14–16]; diatoms and larger dinoflagellates (>20 μm), were dominant on the shelf, while dinoflagellates were dominant off shelf [14, 16]. An exception was the diatom *Cheatoceros*, which was found to be dominant off shelf in spring [14] and summer [15], implying there are also important seasonal factors to consider. For example, during spring and summer, phytoplankton communities were structured by different levels of stratification and mixing between the shelf break and thermal or salinity fronts [14, 15]. In contrast, during autumn weakened stratification, increased nutrient availability and stronger mixing caused by seasonal winds and stronger heat loss to the atmosphere were considered to be a major driving force underlying differences in phytoplankton communities [16].

Even the most recent of the above studies was based on observations collected over a decade ago from a single transect comparing shelf and oceanic stations with no data collected near the shelf edge [16]. This study is the first to compare data from multiple cross shelf transects with data collected from the potentially important shelf edge. This study will give a novel insight about differences and similarities of phytoplankton communities on both latitudinal and longitudinal gradients in the NWES. The aim of the study is to draw conclusions of the structuring forces and environmental drivers of phytoplankton occurrence on the Malin and Hebridean shelves and to provide some much needed baseline data for the MSFD. This was achieved through a systematic set of transects that sampled phytoplankton and environmental data shelf, shelf break, and oceanic water across a range of different latitudes on the NWES. Understanding phytoplankton dynamics and their environmental controls is crucial to allow further work on the effect of environmental changes on the biological carbon pump provided by phytoplankton. Moreover, information on the connectivity and separation of phytoplankton on the shelf edge can be used to support modelling work on advection of harmful phytoplankton in the area.

## Methods

**Fig 1:**
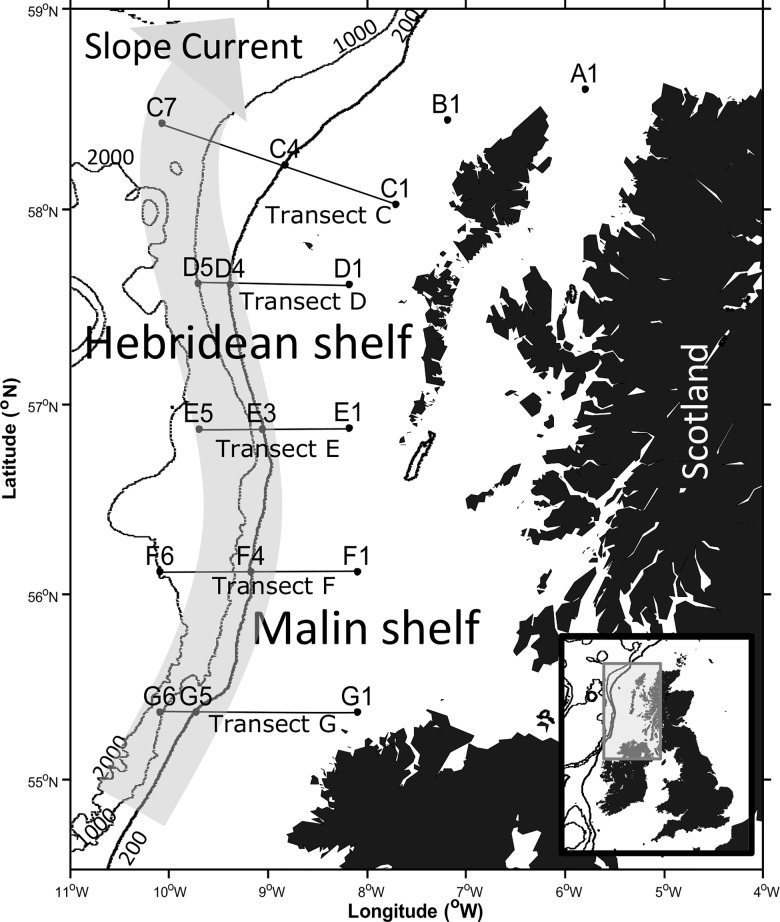
Cruise track and sampling sites of the RRS Discovery cruise DY017 to the N.W. European shelf break with the path of the ESC marked in grey. Full details of stations where net samples were taken are provided in Table 1.

Samples were collected on 7 transects across the shelf, shelf edge and open ocean during RRS *Discovery* cruise DY017 to the Hebridean (A1, B1 transect C-D) and the Malin (transect E-G) Shelves between the 23^rd^ October 2014 and 3^rd^ November 2014 (Fig 1). Weather conditions were stormy prior to and during the cruise inhibiting sampling at some of the planned stations. A total of 17 sites were sampled, including 7 shelf, 5 shelf break and 5 oceanic stations (Table 1).

**Table 1:** Details of stations where net samples were taken and maximum depth for each net tow.

Station	Position	Date	Time	Water depth (m)	Latitude	Longitude	Depth of Net tow (m)
A1	Shelf	23.10.14	11:06	105	58.60	-5.80	50
B1	Shelf	27.10.14	03:52	75	58.44	-7.19	50
C1	Shelf	27.10.14	10:00	75	58.02	-7.71	70
C4	Shelf break	27.10.14	22:08	190	58.22	-8.83	100
C7	Oceanic	28.10.14	21:24	1860	58.43	-10.07	100
D5	Oceanic	29.10.14	05:13	955	57.62	-9.70	80
D4	Shelf break	29.10.14	10:13	190	57.61	-9.38	60
D1	Shelf	30.10.14	01:47	115	57.61	-8.18	100
E1	Shelf	30.10.14	08:51	130	56.87	-8.18	60
E3	Shelf break	30.10.14	17:07	190	56.86	-9.05	50
E5	Oceanic	31.10.14	13:45	1850	56.87	-9.70	70
F6	Oceanic	01.11.14	18:12	1980	56.12	-10.09	100
F4	Shelf break	02.11.14	06:26	185	56.11	-9.17	50
F1	Shelf	02.11.14	17:46	100	56.11	-8.10	80
G1	Shelf	02.11.14	23:52	60	55.37	-8.09	60
G5	Shelf break	03.11.14	09:51	190	55.36	-9.73	70
G6	Oceanic	03.11.14	17:36	1125	55.36	-10.10	100

Densities of microphytoplankton in Niskin bottle samples were below the detection limit of 50 ml Utermöhl method microscopy analyses. Phytoplankton abundance data presented is therefore based on cells collected via a 20 μm phytoplankton net haul to the bottom of the mixed layer depth which varied between 50 m and 100 m at each station (Fig 1, [15]). The total volume of filtered seawater was calculated as the volume of a cylinder: Volume = π x r^r^ x h with r being the radius of the net opening and h the depth of the net haul. Net collected samples were stored in 60 ml brown plastic bottles rinsed with seawater. Samples were immediately fixed with 1 ml acidic Lugol's Iodine solution and subsequently settled for at least 22 hours at room temperature in 10 ml or 25 ml settling chambers. Cells were identified under 200x magnification using a Zeiss Axiovert 100 inverted light microscope. Species counts from net hauls were divided by the filtered volume and calculated as cells per litre to account for different depth sampled by the haul. Species and genera present at greater than 1 cell l^-1^ x10^2^ were *Tripos furca*, *T*. *fusus*, other *Tripos* spp., *Prorocentrum* spp., *Dinophysis* spp. and *Pseudo-nitzschia* spp. (N.B. we used the current taxonomic convention for describing species formally recognised as belonging to the *Ceratium* genus; [17]). Cell counts were calculated as cells l^-1^ to account for differences in the total sampled volume. Phytoplankton cell densities were found to be very low at the time of the study, with an average of 8 phytoplankton cells per litre. This is consistent with the low number of phytoplankton cells found in Niskin bottle samples. Cell counts were fourth root transformed prior to statistical analysis to down weight high species abundance [16].

Data on chlorophyll fluorescence, turbidity, light attenuation, temperature, salinity and density were collected *in situ* during CTD casts. Chlorophyll was measured from three different depths at each station by filtering 500 ml through a 47 mm glass fibre filter (Whatman GF/F). Filters were stored in the dark at -20°C prior to extraction. Filters were thawed overnight in the dark with 8 ml of 90% acetone at 4°C and subsequently sonicated for 1 min and centrifuged for 5 min at 3000 rpm. Chlorophyll a was measured with a Turner trilogy fluorometer. The fluorometer was calibrated using a chlorophyll a standard (spinach extract) at concentrations from 1 to 5 μg l^-1^, verified using a scanning spectrophotometer (Nicolet evolution 300, Thermo Electron Corporation, Cambridge, UK). Chlorophyll measurements were used to calibrate CTD fluorescence for chlorophyll using the linear relationship Chlorophyll = 0.14225 + 0.710xFluorescence (p < 0.001) that was evident between measured chlorophyll and CTD fluorescence.

Samples for nitrate, silicate and phosphate concentrations were collected from Niskin bottle deployments associated with the CTD casts and measured on board using a Skalar *San+* segmented flow autoanalyser following methods described by Kirkwood [18]. Environmental data were standardised using Wisconsin double standardization as the data were recorded in different units and would not be comparable otherwise [19, 20]. Moreover environmental data was averaged over the depth sampled by the parallel phytoplankton net deployment to provided average values for environmental parameters in the mixed surface layer.

Analysis of Similarity (ANOSIM) was calculated using the MATLAB toolbox “fathom”. All other analyses were undertaken in R using the packages “vegan” and “MASS”. Non-metric multidimensional scaling (nMDS) and principal component analysis (PCA) were used for biological and environmental data respectively. Environmental data was then fitted to the biological ordination using the R vegan function “envfit()”, calculating r^2^ to assess the goodness of fit for a linear model [21].

## Results

### Overview

**Fig 2:**
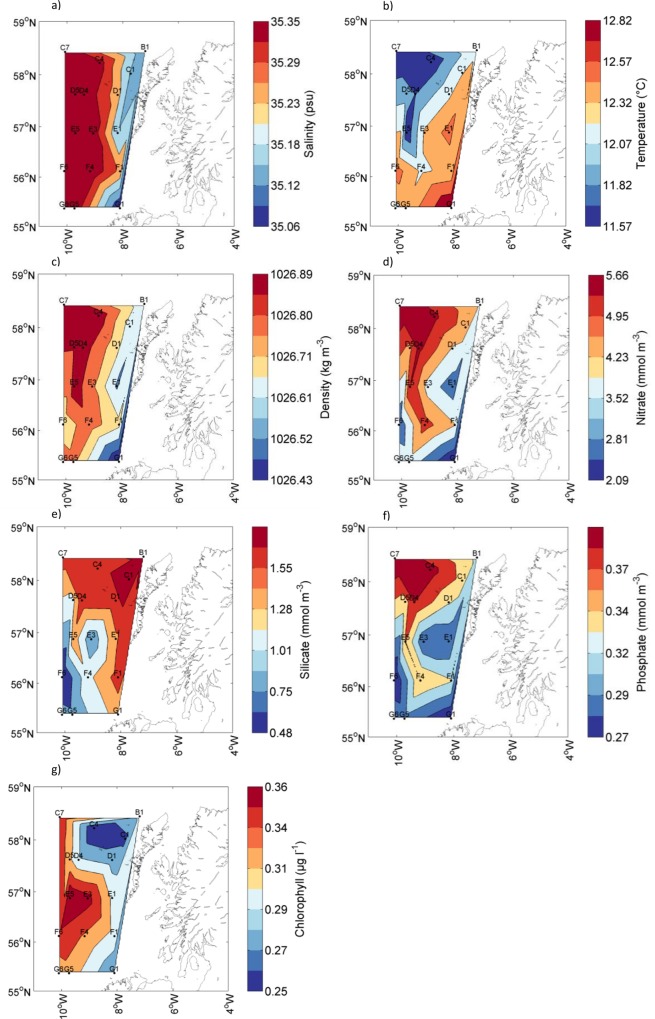
Average surface concentration of the top 20 m for a) Salinity, b) Water temperature, c) Density, and for the top 50 m for d) Nitrate, e) Silicate, f) Phosphate and g) Chlorophyll from fluorescence.

Due to a large number of data points during depth profiling the average value for the top 20 meters for CTD data and top 50 meters for nutrient data is presented to allow a comparison of the surface waters between sites (Fig 2). The surface salinity at shelf stations varied from 34.73 (A1) to 35.3 (D1) but had a narrower range of 35.51 to 35.57 at all shelf break and oceanic stations (Fig 2A). The highest recorded surface temperature was 12.9°C at the southernmost shelf station G1 and temperature decreased both northwards on shelf and westwards towards oceanic stations with the minimum recorded surface temperature of 11.6°C measured at the north-westerly oceanic station C7. Average density of the top 20 m was highest (1026.98 kg m^-3^) in the cool, saline water at the shelf break and oceanic waters. Water densities were lower in the less saline shelf waters and the warmer waters in the south of the study area (Fig 2C). Nitrate and phosphate distributions displayed the reverse pattern to temperature, being highest in the north and along the shelf break (Fig 2D and 2F). Silicate concentrations were also highest in the northernmost stations but concentrations were generally patchy over much of the shelf (Fig 2E). The pattern of surface chlorophyll fluorescence did not coincide with any of the nutrient distributions with highest chlorophyll fluorescence at stations E3 and E5 and lowest values for station C4 (Fig 2G).

**Fig 3:**
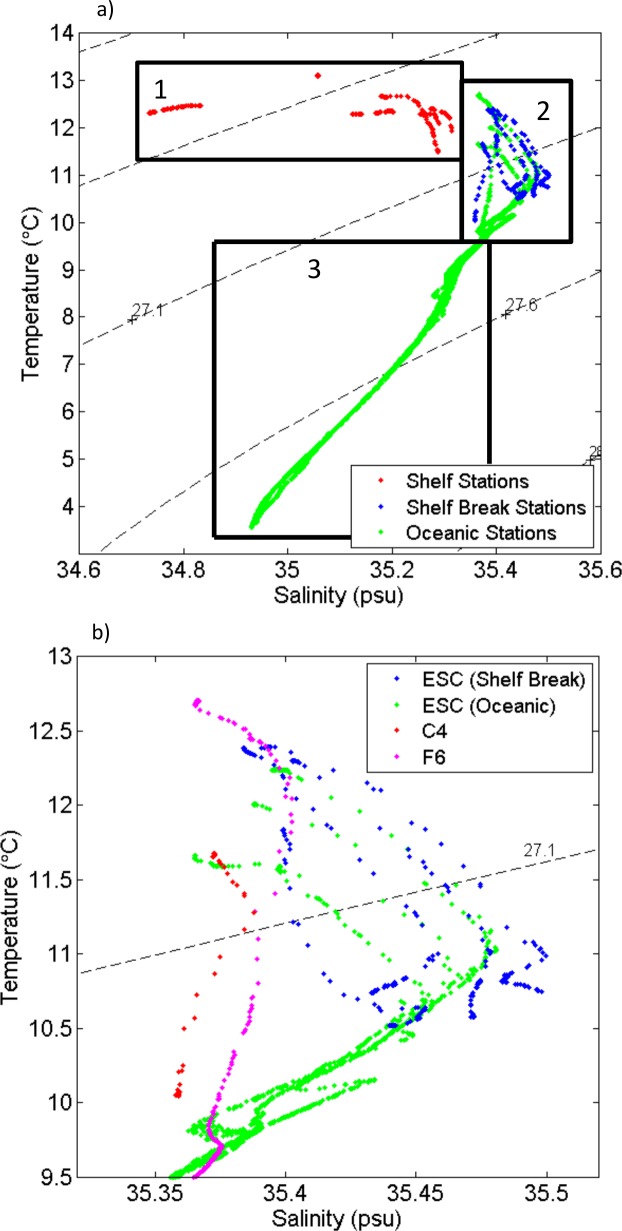
Water temperature and salinity diagram with isopycnals (dashed lines) of a) all stations. Box 1 shelf water, box 2 surface water for shelf break and oceanic stations, box 3 water below 200 m at oceanic stations. b) detailed view of box 2 with stations with an ESC signature marked in blue (shelf break stations) and green (oceanic stations) and stations outside the ESC in red (C4) and magenta (F6).

The different water masses present in the study area were identified from their salinity and temperature properties (Fig 3). Shelf stations had characteristic water properties with lower salinities and higher temperature (Fig 3A, box 1). The highest salinities were found at shelf break stations and within the top 200 m of oceanic stations. Stations C7, D4, D5, E3, E5, F4, G5 and G6 had an increased salinity over 35.4 psu, which is characteristic for the ESC (Fig 3A, box 2, Fig 3B, [22]). Shelf break station C4 and oceanic station F6 are located east and west of the slope current respectively. Waters below 200 m at oceanic stations revealed evidence of several different water masses. Water below the high salinity waters of the ESC showed characteristics of the East North Atlantic Water with water temperature ranging from 8°C to 10°C and salinity from 35.22 to 35.53. At an intermediate depth between 600 and 1200 m Wyville Thomson Overflow water flowing into the region from the north and gradually mixed with the deep Labrador Sea water entering from the south, with characteristically low water temperatures below 4°C and salinities around 34.9 ([23], Fig 3A, box 3).

Species counts at station C4 were at least an order of magnitude lower than for other stations, with less than 1 cell l^-1^, while station G1 had the highest cell count with 18 cells l^-1^ (Table 2). *Tripos fusus*, followed by *Tripos furca*, were the most abundant species and both were present in all samples together with other *Tripos* spp. (Table 2). *Dinophysis*, *Pseudo-nitzschia* and *Prorocentrum* were less numerous and present in all shelf stations but were absent or rare (less than 1 cell l^-1^) in shelf break and oceanic stations (Table 2).

**Table 2:** Cells l^-1^ x10^2^ and percentage of the genera/species present at each station.

St	*T*. *fusus*		*T*.*furca*		*Tripos spp*. *(other)*		*Proro-centrum*		*Pseudo-nizschia spp*.		*Dinophysis spp*.	
	count	%	count	%	count	%	count	%	count	%	count	%
Shelf Stations												
A1	7	5.2	13	9.4	21	14.9	71	50.6	12	8.7	16	11.3
B1	17	5.3	44	14.1	97	30.8	107	34.2	33	10.5	16	5.2
C1	17	6.7	28	11.3	122	48.7	51	20.2	9	3.5	24	9.6
D1	30	11.7	7	2.7	157	62.1	33	12.9	7	2.7	20	8.0
E1	243	27.0	13	1.4	453	50.3	15	1.7	151	16.7	25	2.8
F1	422	57.9	12	1.6	61	8.4	15	2.0	182	24.9	38	5.2
G1	813	46.2	2	0.1	194	11.0	13	0.8	646	36.7	92	5.2
Shelf break Stations												
C4	13	66.4	6	30.0	<1	1.4	0	0.0	<1	2.2	0	0.0
D4	335	60.1	146	26.2	68	12.2	3	0.5	2	0.4	4	0.6
E3	828	52.8	495	31.5	243	15.5	<1	0.0	0	0.0	2	0.1
F4	501	48.1	314	30.1	225	21.6	1	0.1	0	0.0	2	0.2
G5	677	54.7	348	28.1	211	17.0	0	0.0	1	0.1	1	0.1
Oceanic stations												
C7	654	56.7	377	32.7	122	10.6	0	0.0	0	0.0	<1	0.0
D5	639	52.6	448	36.9	128	10.5	0	0.0	1	0.1	0	0.0
E5	589	50.9	467	40.3	99	8.6	<1	0.0	1	0.1	1	0.1
F6	395	42.0	392	41.6	154	16.3	0	0.0	0	0.0	1	0.1
G6	403	50.3	247	30.9	150	18.7	0	0.0	1	0.1	<1	0.0

### Shelf Environment and Associated Phytoplankton

**Fig 4:**
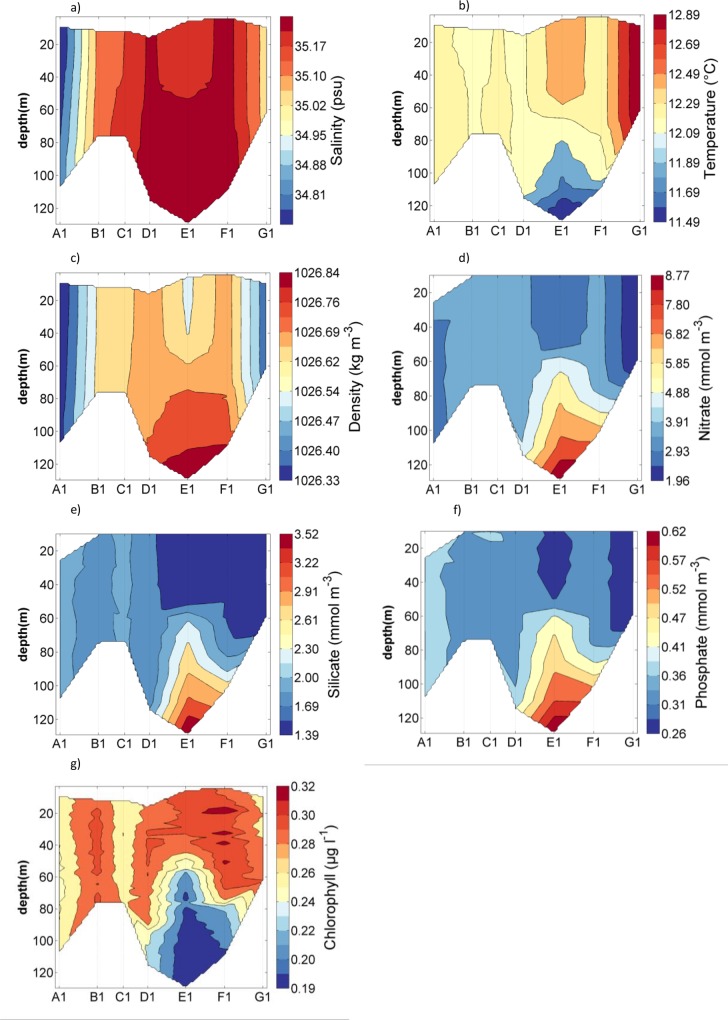
Contour plots for on shelf stations (whole water depth) for a) Salinity, b) Water temperature, c) Density, d) Nitrate, e) Silicate, f) Phosphate and g) Chlorophyll from fluorescence.

Based on the hydrography and topography at sampling locations, stations were divided into shelf (between 80 m and 120 m depth), shelf break (around 200 m depth) and oceanic stations (1000 m to 2000 m depth). Density indicated that shelf stations were well mixed with the exception of E1, where density increased with depth (Fig 4C). Salinity was lowest throughout the water column at station A1 (34.9) and highest at stations D1, E1 and F1 (Fig 4A). Water temperature increased from around 12.1°C at the northern stations to 12.9°C at the southern station G1. Temperature for the stratified station E1 decreased from 12.7°C at the surface to 11.5°C below 120 m (Fig 4B and 4C). Nutrient concentrations and chlorophyll fluorescence showed opposing trends (Fig 4D–4G); throughout the water column for all stations, except station E1, chlorophyll fluorescence was constant around 0.2 to 0.3 μg l^-1^ and nutrients were lowest with 2 to 4 mmol NO_3_ m^-3^, 1.4 to 2.3 mmol Si m^-3^ and 0.26 to 0.4 mmol PO_4_ m^-3^. As chlorophyll fluorescence decreased to near zero below 80 m at station E1, nutrient concentrations increased to 9.74 mmol NO_3_ m^-3^, 3.83 mmol Si m^-3^ and 0.67 mmol PO_4_ m^-3^ (Fig 4D–4G).

All identified phytoplankton species were found at all shelf stations, however, the dominant species/genus changed along the north-south gradient (Table 2). *Prorocentrum* was dominant in the northern shelf stations, making up to 50% and 34% of the population at station A1 and B1 respectively, but decreased rapidly to less than 1% at station G1 in the south. *T*. *fusus* showed the opposite trend, accounting for 5% at A1 but being the most dominant species at the southern stations F1 and G1. At stations C1, D1 and E1 other *Tripos* species were most dominant. *T*. *furca* decreased from around 10% at northern stations to nearly zero (0.1%) at station G1. The pattern for *Pseudo-nitzschia* was less clear with community proportions that varied from 3% at station D1 to 37% at station G1. *Dinophysis* had the lowest overall presence representing 11% of the community at station A1 and 3% at station D1 (Table 2).

### Shelf Break Environment and Associated Phytoplankton

**Fig 5:**
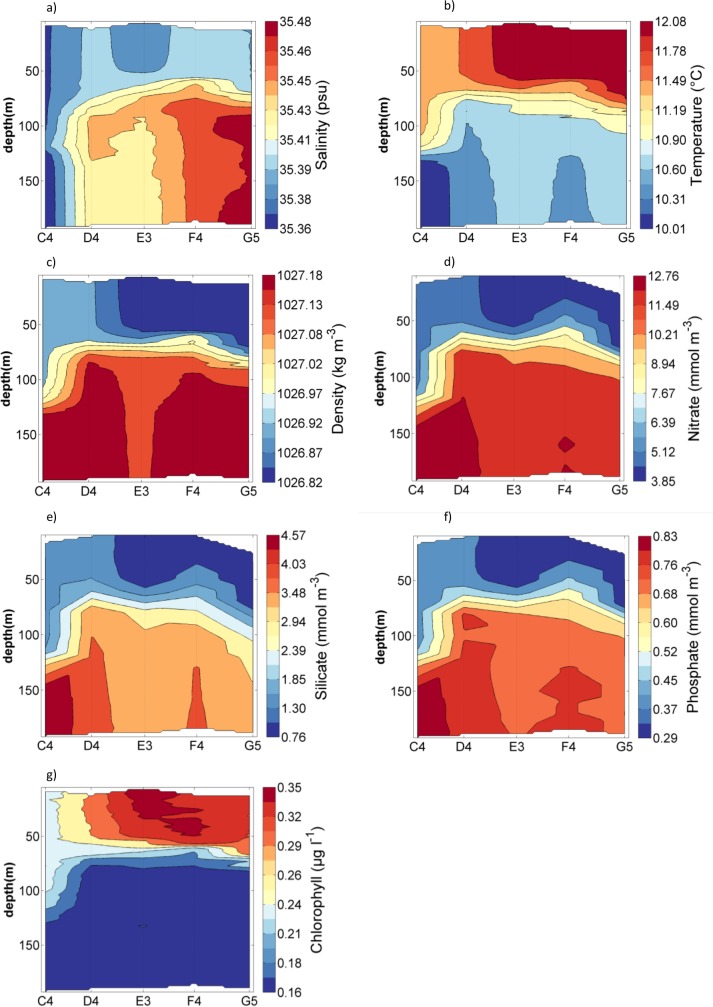
Contour plots for shelf break stations (whole water depth) for a) Salinity, b) Water temperature, c) Density, d) Nitrate, e) Silicate, f) Phosphate and g) Chlorophyll from fluorescence.

At shelf break stations salinity, water temperature and density profiles suggested a surface mixed layer with a depth of 60–100 meters (Fig 5A–5D). The high salinity core with salinities over 35.4 which is characteristically for the slope current was found below 50 m at stations D4 to G5. Station C4 was the only shelf break station to not reach salinities over 35.4, indicating that the station was located east of the slope current. Station C4 also displayed the lowest water temperatures and deepest mixing depth compared to other stations, which might be linked to its position outside the ESC and the lack of stratification caused by salinity. Both surface and deep water temperature increased with decreasing latitude. Nutrient concentrations (Fig 5D–5F) were low throughout the surface mixed layer with concentrations of 3.8 mmol NO_3_ m^-3^, 0.76 mmol Si m^-3^ and 0.29 mmol PO_4_ m^-3^, but concentrations rapidly increased below the mixed layer depth to 14.03 mmol NO_3_ m^-3^, 5.12 mmol Si m^-3^ and 0.91 4 mmol PO_4_ m^-3^. Chlorophyll fluorescence was between 0.13 and 0.32 μg l^-1^ in the surface layer, without any visible maxima, and declined rapidly to near zero concentrations at depth (Fig 5G). The phytoplankton assemblage was dominated by *Tripos* with an average of 0.4% of phytoplankton accounted for by non *Tripos* cells (Table 2). *T*. *fusus* was the dominant species at all shelf break stations and accounted for an average of 56% of cells, followed by *T*. *furca* (29%, Table 2).

### Oceanic Environment and Associated Phytoplankton

**Fig 6:**
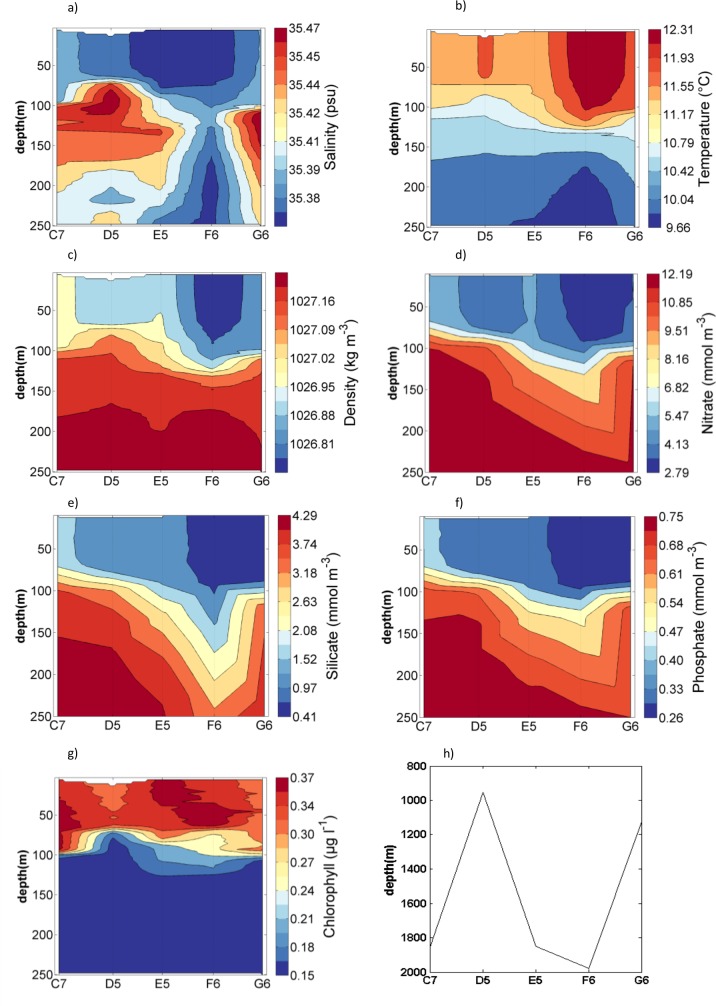
Contour plots for shelf break stations (top 250 m) for a) Salinity, b) Water temperature, c) Density, d) Nitrate, e) Silicate, f) Phosphate, g) Chlorophyll from fluorescence and h) water depth.

The water depth at oceanic stations varied between 1000 and 2000 m (Fig 6H). Similar to shelf break stations, a surface mixed layer depth of 60–100 meters was evident from salinity, water temperature and density profiles at oceanic stations (Fig 6A–6D). Increased salinities below 100 meters indicated that the path of the slope current included all oceanic stations except F6 that lay west of the slope current (Figs 6A and 1). Nutrient concentrations were similar to shelf break stations with low concentrations throughout the surface mixed layer of 2.79 mmol NO_3_ m^-3^, 0.41 mmol Si m^-3^ and 0.26 mmol PO_4_ m^-3^, and increased concentrations of up to 13.54 mmol NO_3_ m^-3^, 4.85 mmol Si m^-3^ and 0.82 mmol PO_4_ m^-3^ below the mixed layer depth (Fig 6D–6F). The phytoplankton community was similar to shelf break stations with *T*. *fusus* being the most common species accounting for an average of 51% of cells at each station, followed by *T*. *furca* which accounted for 36% of cells (Table 2). The remaining percentage consisted of mainly other *Tripos* species with less than 0.1% accounted for by non *Tripos* cells (Table 2).

### Intra-Regional Variability

**Fig 7:**
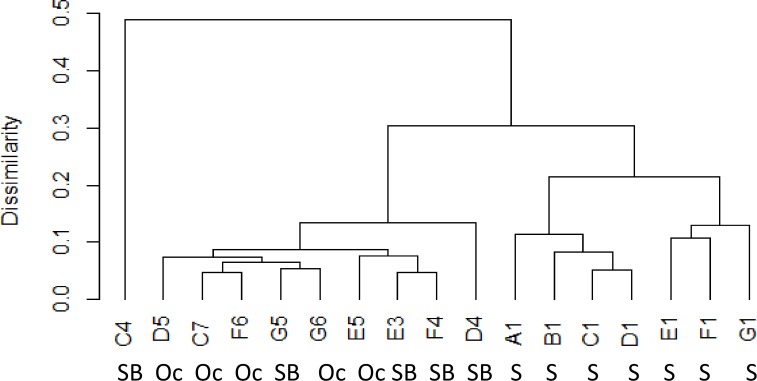
Dendrogram of the average Bray-Curtis distance between sites and site groupings based on phytoplankton count data (S = shelf, SB = shelf break, Oc = oceanic station)

**Fig 8:**
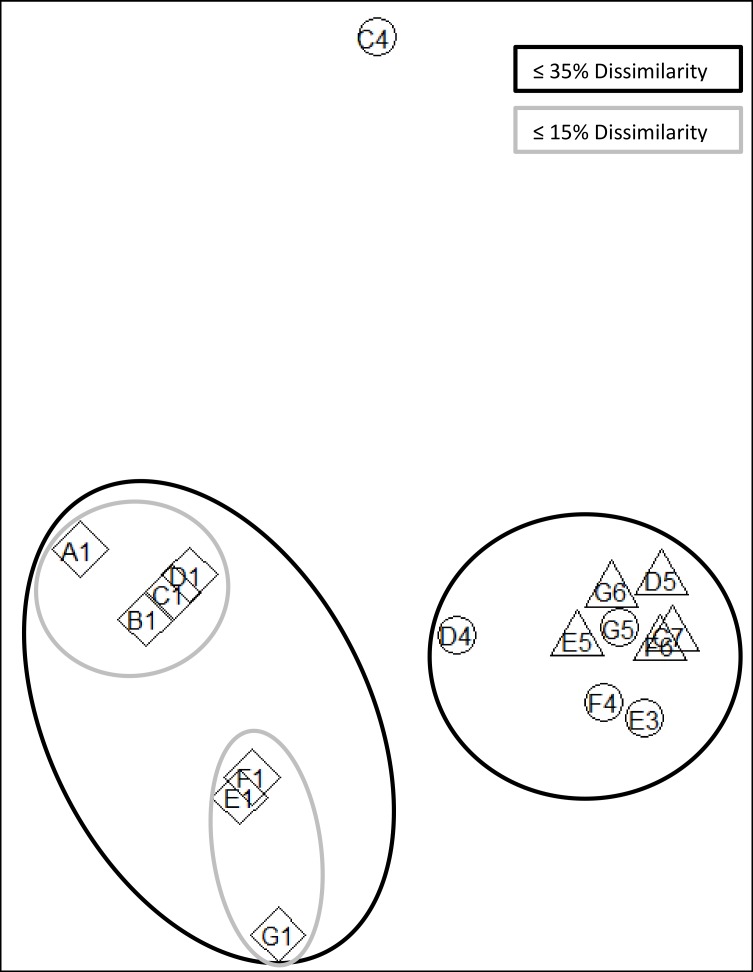
Non-metric multidimensional scaling (nMDS) ordination of fourth root transformed species data at each site sampled during cruise DY017 to the N.W. European shelf. The stress is 0.036 indicating that ordination is representable for real patterns rather than randomness in the data. (Triangle = oceanic stations, circle = shelf break stations, diamond = shelf stations).

Phytoplankton community at the shelf, shelf break and oceanic sites were significantly different (ANOSIM, p = 0.001, r = 0.5481). Shelf stations were significantly different to shelf break stations (ANOSIM, p = 0.001, r = 0.6793) and oceanic stations (ANOSIM, p = 0.001, r = 0.9779) while shelf break and oceanic stations were not significantly different to one another (ANOSIM, p = 0.25, r = 0.0720). Different grouping was visualised by a dendrogram of the Bray-Curtis distance (Fig 7) and non-metric multidimensional scaling (nMDS) of phytoplankton counts (Fig 8). In the nMDS diagram increasing distance between sites represents higher dissimilarities between the species composition at each site (Fig 8). Shelf break and oceanic stations were no more than 35% dissimilar to each other with the exception of station C4, which was around 50% dissimilar to any other sampled station (Figs 7 and 8). Ordination of shelf stations showed a stronger response to the north-south gradient than to the cross-shelf gradient between shelf and shelf break/oceanic stations. Around 20% dissimilarity were observed between northern stations A1-D1 and southern stations E1-G1 with the northernmost station A1 being the least similar to the southernmost station G1 within the on shelf group (Fig 8).

**Fig 9:**
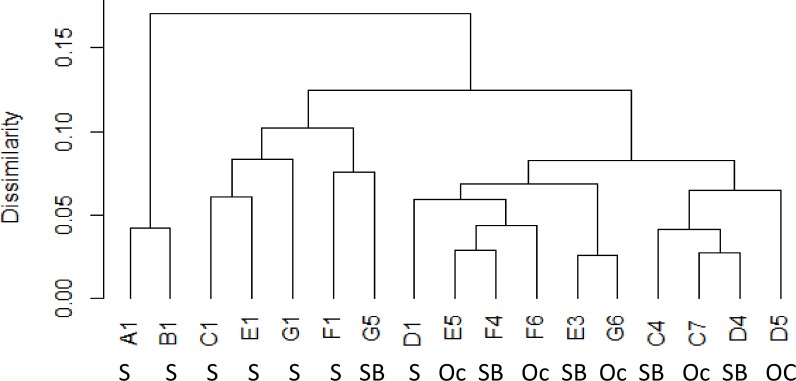
Dendrogram of the average Bray-Curtis distance between sites and site groupings based on environmental data (S = shelf, SB = Shelf break, Oc = oceanic station).

**Fig 10:**
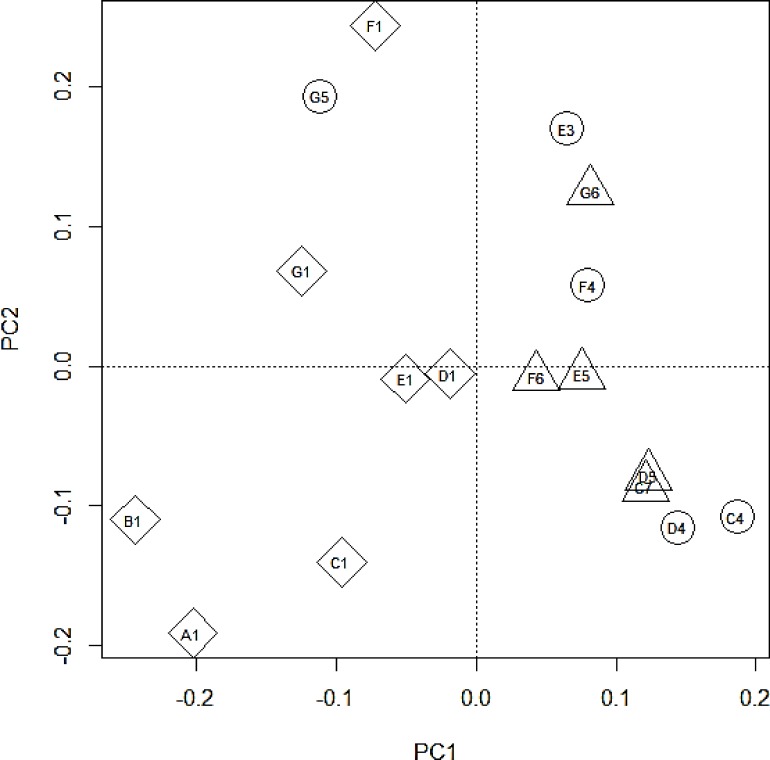
Principal component analysis (PCA) ordination of Wisconsin standardised environmental data at each site sampled. (Triangle = oceanic stations, circle = shelf break stations, diamond = shelf stations).

Based on their physical (salinity, water temperature, density) and chemical (nitrate, phosphate, silicate) properties there was no statistically significant difference between groups (ANOSIM, p = 0.29, r = -0.06) with all sites being no more than 20% dissimilar (Figs 9 and 10). However, the similarity analysis indicated a grouping of shelf stations and a separate grouping of the remaining stations with the exception of station G5 (grouped with shelf stations) and station D1 (grouped with oceanic/ shelf break stations) (Fig 9). Increasing distance between sites in the PCA plot represents higher dissimilarity in the chemical and physical properties of the site. A total of 86.88% of variance in the data could be explained by the two dimensional representation. 59.47% of it was explained by the principal component 1 (PC1). Shelf stations were separated from other stations along the PC1 axis with all shelf stations (including G5) having a negative PC1 value while all other sites had a positive PC1 value (Fig 10). An additional 27.42% of the variance in the data was explained by the principal component 2 (PC2) Along the PC2 axis the north-south gradient was visualised with northern transects A-D having a negative PC2 value and southern transects E-G having positive or close to zero PC2 values (Fig 10).

**Fig 11:**
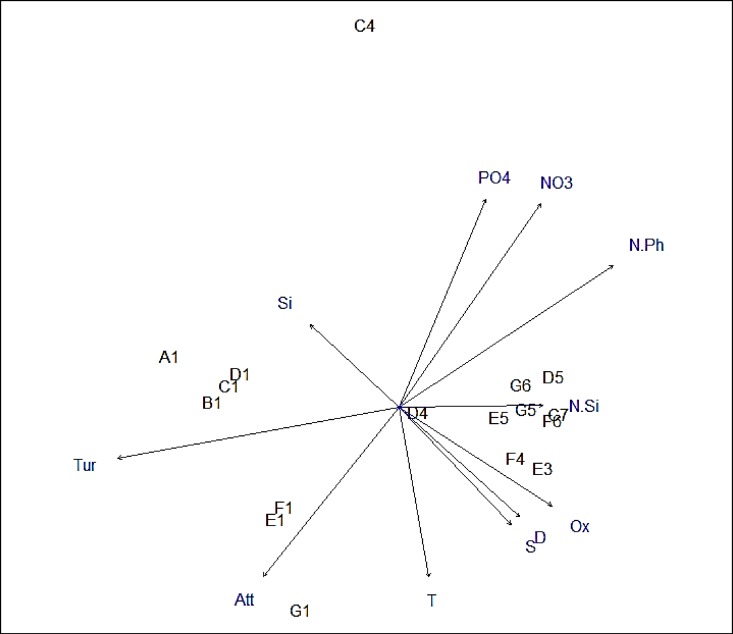
Vectors of environmental factors plotted on the nMDS ordination of species composition indicating the relation between environmental factors and species composition. The value of an environmental factor increases towards the direction of the arrow while significance is indicated by arrow length (Tur = Turbidity, Att = light attenuation, T = temperature, S = salinity, D = density, NO3 = nitrate, PO4 = phosphate, Si = silicate, N.Ph = nitrate to phosphate ratio, N.Si = Nitrate to silicate ratio).

Vectors of environmental variable were fitted to the nMDS ordination of phytoplankton data to visualise the relationship between environmental data and phytoplankton composition and distribution (Fig 11). The length of the environmental vector is proportional to the correlation between the environmental factor and the phytoplankton ordination. The arrow indicates the direction towards which the environmental variable increases (Fig 11). The significance of the relation between an environmental driver and the phytoplankton ordination is determined by calculating their goodness of fit (r^2^) and their p-value (Table 3). Turbidity, nitrate and phosphate concentrations, the nitrate to phosphate ratio and light attenuation had a statistically significant relation to phytoplankton community structure (Fig 11, Table 3). Turbidity and light attenuation were highest on the shelf while nitrate, phosphate and the nitrate to phosphate ratio were highest at oceanic and shelf break stations. Turbidity was also higher in northern shelf stations than southern shelf stations, opposite to light attenuation. However, the difference between shelf and oceanic stations was higher for turbidity than for light attenuation.

**Table 3:** Environmental factors ordered by the significance of their relationship to phytoplankton ordination, with r^2^ value for goodness of fit and their significance, with p being significant for p ≤ 0.05.

Environmental driver	r^2^	P	
Turbidity	0.7408	0.0001	Significant at
Nitrate/Phosphate ratio	0.5981	0.0015	P < 0.005
Nitrate	0.5614	0.0029	
Phosphate	0.4639	0.0104	Significant at
Attenuation	0.4250	0.0184	P < 0.05
Oxygen	0.2989	0.0792	Not significant
Temperature	0.2682	0.1125	P > 0.05
Density	0.2402	0.1427	
Salinity	0.2374	0.1459	
Nitrate/ Silicate ratio	0.1875	0.2304	
Silicate	0.1340	0.3706	

## Discussion

### Hydrodynamics

The ESC flows along the steep continental slope between 200 m and 2000 m depth and is characterised by a high salinity core with salinities over 35.42 [22]. In this study salinities higher than 35.4 were detected at stations C7, D4, D5, E3, E5, F4, G5 and G6 (Fig 3). Stations C4 and F6, with salinities generally below 35.4, were located to the east and west of the slope current respectively. Station F6 was 2000 m deep with the 2000 m isobaths indicating the end of the continental slope. Station C4 was located at 200 m, the same approximate depth as the shelf break stations, however, drifters released along the continental slope showed dispersion of the ESC towards deeper waters around this location due to its irregular bathymetry [24]. Shelf stations were located east of the slope current with salinities below 35.32, caused in part by terrestrial fresh water input. Lower temperatures at shelf break and oceanic stations are most likely caused by heat loss to deeper waters as stratification weakens over autumn.

The ESC is considered to act as a boundary between the shelf and oceanic water with limited water exchange from wind driven surface eddies [10]. However, increased cross shelf water exchange can often be found when the bottom slope increases, causing leakage of high salinity slope water onto the shelf [25]. Such leakage can potentially transport oceanic phytoplankton into shelf waters [15]. Recent model studies also suggested that intrusion from slope current water might provide a seed population for harmful phytoplankton to the shelf [26]. In this study shelf stations had distinctly low salinities, giving no indication of cross shelf edge exchange despite strong seasonal winds. Phytoplankton communities were also significantly different at shelf stations compared to shelf break and oceanic stations, suggesting that there was little exchange of phytoplankton during the study period (Figs 7 and 8). The potentially toxin producing genera *Dinophysis* and *Pseudo-nitzschia* were found in much higher numbers at shelf sites than sites within the ESC suggesting that the ESC did not provide a seed population of harmful phytoplankton to the shelf during the study period. Phytoplankton at station F6 were very similar to other oceanic and shelf break stations despite being west of the slope current. The phytoplankton community at C4 was most dissimilar to other shelf break and shelf stations, but also exhibited little similarity the shelf stations indicating a different, but unknown, structuring factor at this site.

The ESC and parallel running coastal currents are known to play an important role in the transport of phytoplankton; e.g. a potentially harmful patch of *Dinophysis* was observed to travel with the same speed and direction as the jet like coastal current along southwest Ireland [27], while a major bloom of the fish killing *Karenia mikimotoi* is thought to have been advected northwards along the west Scottish coast [26, 28]. In this study nMDS of phytoplankton showed that northern and southern shelf stations were about 20% dissimilar, suggesting that limited connectivity between stations allowed for the formation of different communities along the latitudinal gradient. However northern and southern shelf stations were still more similar to each other than to shelf break/oceanic stations. This suggests there was a higher degree of connectivity and exchange of phytoplankton on the south to north to south gradient on the shelf than along the longitudinal gradient. In contrast, there was no latitudinal gradient in the community similarity index for shelf break or oceanic stations, which suggests a stronger exchange of phytoplankton along the shelf edge by the slope current.

### Phytoplankton Counts and Chlorophyll Fluorescence

Phytoplankton communities were significantly different in terms of species composition, richness and abundance between shelf stations and shelf break/oceanic stations (Figs 7 and 8). Diatoms exhibited their maximum abundance on the shelf as previously found on the Malin shelf in spring and summer [14] and autumn [16]. However, in contrast to previous studies, dinoflagellates were more numerous than diatoms on the shelf (Table 2). Phytoplankton blooms are often characterised by an early diatom dominance which is replaced by dinoflagellate dominance in later bloom stages. In this study, samples were taken in late autumn and dinoflagellate dominance together with generally low cell numbers suggested that samples were taken at the end stages of the autumn bloom or after bloom termination. At shelf break and oceanic stations diatoms were absent or accounted for less than 3% of the total species count. Dinoflagellates were previously found to dominate oceanic waters adjacent to the shelf, however, some diatoms were also found to be more numerous at oceanic stations [14, 16]. Here, the dinoflagellate *Tripos* dominated all oceanic and shelf break stations while shelf stations supported a greater diversity, with *Prorocentrum* spp. dominant at one station and *Dinophysis* spp. present at all shelf stations. The potentially toxin producing genera *Dinophysis* and *Pseudo-nitzschia* were found in low cell concentrations and might not pose an immediate threat to human health. Even low cell numbers can provide a seed population for subsequent development of harmful blooms once growth conditions improve as suggested by Raine [29] and Davidson et al. [28], although this is less likely in the late autumn conditions of this study.

All species identified in this study are common members of the phytoplankton community of the Malin and Hebridean Shelfs [14, 16, 30]. Continuous Plankton Recorder (CPR) data from tracks at the northern edge of the study area suggest that *Tripos* often reach their maximum numbers in autumn [30]. While Hinder et al. [31] found that dinoflagellates are generally declining in abundance in the North East Atlantic, with *Tripos* and *Prorocentrum* having declined noticeably in the early 2000s compared to previous decades [31], our results are in accordance with Johns [30], who found *Tripos* to be the dominant genus in late October 2014.

Under suitable growth conditions phytoplankton can form early autumn blooms. Chlorophyll concentrations of 2.3 μg l^-1^ were reported on the Malin Shelf by Fehling et al. [16], which is about 10 times higher than chlorophyll concentrations recorded in this study. Our values are however consistent with other studies, for example, a late autumn bloom in November 1985 on the Armorican and Celtic shelves caused by sunny and calm conditions reached chlorophyll concentrations of up to 0.7 μg l^-1^ in the shelf break region and slightly lower concentrations in adjacent oceanic waters [32]. In this study chlorophyll increased slightly over the shelf break and oceanic waters within the path of the slope current with chlorophyll concentrations being about half of those observed by Garcia-Soto and Pingree [32]. Differences in chlorophyll concentrations might be partly linked to differences in picophytoplankton; however picophytoplankton was not accounted for here or in previous studies of the area [14–16, 32]. CPR measurements in the area showed that there is high inter-annual variability in microphytoplankton cell numbers in autumn with cell abundance occasionally being close to zero [30], which could explain discrepancies in chlorophyll measurements between studies.

One explanation for this inter-annual variability might be differences in timing and intensity of seasonal storms. Generally storms increase mixing, which can in turn increase nutrient availability in the surface layer and enhance phytoplankton growth [33, 34], although they can also delay bloom formation on some occasions due to the increase of suspended particulate matter in surface waters [35]. Few studies have measured the immediate phytoplankton response in situ in shelf or oceanic waters to heavy storms. Malone et al. [36] found that phytoplankton would decrease by 70% as an immediate storm response, followed by a sharp increase in productivity as newly available nutrients were utilised. Similarly, primary production showed a sharp decline following a storm event in the Celtic Sea [9]. There was no noticeable decrease in phytoplankton cell numbers during the sampling period, despite the presence of heavy winds. Strong winds were already affecting the study area prior to the cruise and the low cell numbers found here might be the result of a decline in phytoplankton due to heavy winds prior to the study.

### Nutrients and Nutrient Ratios

Nutrient concentrations were lowest in surface waters, where chlorophyll fluorescence was above 0.2 μg l^-1^. Below the mixed surface layer fluorescence rapidly decreased to close to zero values, while nutrient concentrations displayed their maximum values (Figs 4 and 6). This pattern suggested that uptake by phytoplankton was a strong structuring force over nutrient distribution. The same pattern was observed by Fehling et al. [16] in the region in autumn, however, nitrate concentrations in their study were up to 4 mmol m^-3^ lower. Higher nitrate concentrations in this study could be caused by wind driven deep turbulent mixing or low uptake of nutrients by phytoplankton. Both studies found a mixed layer depth of 50 to 100 m, suggesting there was little difference in deep mixing between studies. However, there was a clear difference between studies in the amount of phytoplankton that was present. Chlorophyll concentrations found by Fehling et al. [16] were ten time higher than chlorophyll in this study. Lower phytoplankton numbers would lead to less nitrate uptake and therefore result in higher concentrations of nitrate in this study.

We found differences in nitrate and phosphate concentrations were significantly related to phytoplankton community structure (Fig 11, Table 3). Both nutrients were present at higher concentrations in the surface of oceanic and shelf break stations compared to shelf stations. Phytoplankton counts and chlorophyll fluorescence were also noticeably higher at oceanic and shelf break stations compared to shelf stations, which is consistent with phytoplankton growth being higher due to increased nutrient availability. Silicate responded stronger to the latitudinal gradient than the longitudinal gradient with highest concentrations in the north of the study area. It has been suggested that changes in nutrient ratios can play a crucial role in structuring species composition [37]. In this study the nitrate to phosphate ratio was one of the most significant environmental parameters separating phytoplankton communities. The nitrate to phosphate ratio was between 8:1 (station G1) and 15:1 (stations C7 and E5). Based on the Redfield ratio of 16:1 this would suggest that all sites were limited by nitrate [38]. However, more recent work suggests that the nitrate: phosphate requirements have large variations and can fall between 10:1 to 40:1 and are often below 16:1 without phytoplankton being nitrate limited [39].

Changes in nitrate to silicate ratio can affect the balance between dinoflagellates and diatoms [40]. Several studies suggest that increases in N:Si can shift diatom to dinoflagellate dominance [37]. We found the nitrate to silicate ratio was higher at shelf break and oceanic stations in agreement with results from 2001 that suggested the relative higher availability of silicate per unit nitrate on the shelf was an important driver of phytoplankton community differences [16]. In this study and as reported for observations made in 2001 [16] diatoms had their maximum abundance at shelf stations where more silica per unit nitrate was available. In fact diatom presence was highest (37%) at shelf station G1 where the nitrate to silicate ratio was lowest. While different diatoms have different silicate requirements, studies suggest that at a nitrate to silicate ratio around 2:1 to 3:1, silicate becomes limiting for diatom growth [37]. Here most stations had ratios around 2:1 or higher which could explain the overall dominance of dinoflagellates throughout the study area. The surface silicate concentrations were also generally below the suggested 2μM threshold for diatom dominance [41] with the exception of stations A1 and C1 where silicate concentrations were higher.

### Light Availability

A significant relationship between phytoplankton community and light availability was also evident. Both light attenuation and turbidity were highest on the shelf, indicating reduced light availability for phytoplankton. Such conditions were most likely caused by strong winds leading to mixing and resuspension of sediment at some shallow shelf stations. Chlorophyll fluorescence and cell counts were also generally lower at shelf sites, making it unlikely that cell numbers added significantly to measured turbidity. Turbidity and poor light conditions are often considered to limit phytoplankton growth, however most studies on turbidity focus on estuary environments [42, 43]. Jones and Gowen [44] related light, linked to stratification conditions, to changes in the phytoplankton community at the Malin Shelf. Samples collected in well-lit waters were dinoflagellate dominated while shifts towards a diatom dominated community at sites following a small decrease in light availability. This is consistent with the hypothesis that diatoms are better adapted to higher turbulence, often associated with lower light availability in summer if sufficient nutrients are available [44, 45]. Here we find that diatoms were indeed more numerous at shelf sites with lower light availability, however, as noted above low silicate availability most likely inhibited the development of diatom dominated community.

## Conclusion

We have presented new observations of the phytoplankton community of the Malin and Hebridean continental shelves. We find that stations could be separated by their hydrography into shelf stations and slope current stations with the exception of station C4 at the shelf break which was east of the slope current and oceanic station F6 which was west of the slope current. Consistent with this, phytoplankton communities were broadly divided into two groups; Shelf stations and shelf break/ oceanic stations with the exception of C4, which was the most dissimilar to any other station as a result of the different water mass that was evident at this location. In contrast, the phytoplankton community at station F6 was similar to other oceanic and shelf break stations despite being outside the ESC. Dissimilarities between shelf and shelf break phytoplankton communities supported the hypothesis that the ESC acted as a mixing barrier with low salinities on the shelf confirming limited cross shelf water exchange during the study period. While a latitudinal gradient existed for stations on the shelf, this was not evident for stations within the ESC, indicating a northerly transport of water and phytoplankton within the ESC.

At all stations the phytoplankton community was dominated by dinoflagellates. At shelf break/oceanic stations over 97% of all identified cells belonged to the genus *Tripos*. At shelf break stations phytoplankton diversity was highest and diatoms displayed their maximum abundance representing 37% of the community at station G1. Phytoplankton richness and abundance was generally lower than previously recorded for the Malin shelf in autumn [16], however, it has to be noted that samples in this study were collected later in the autumn season and during a period of heavy winds, which might have had negative impacts on phytoplankton numbers. In this study nutrient concentrations and light conditions had the strongest relation to phytoplankton community differences. The *Tripos* dominated shelf break/ oceanic stations had a higher species abundance also had better light conditions and higher nitrate and phosphate concentrations. Light availability was generally lower at shelf stations. Nitrate and phosphate concentrations were also lower, but more silicate was available for phytoplankton which might explain why diatoms displayed their maximum abundance at shelf stations. The high seasonal and inter-annual variability in both cell numbers and species presence makes it difficult to define a community that would indicate a good ecological status for MSDF monitoring and assessment.

This study presents valuable baseline information to further our understanding of how to determine Good Environmental Status (GES) for the MSFD. The practical and financial difficulties in sampling along and off the shelf edge means that cruise data will have an important role to play in this assessment. Currently a plankton life form approach as described by Tett et al. [46] is being used. The dataset generated here will begin to populate the state space model used in this assessment for the shelf edge and off shelf regions. Data from future cruises will be required to further our understanding of the difference in the phytoplankton community on and off the shelf, if it is changing over time and if changes observed in more coastal stations can also be observed in the offshore. These questions are all key to our understanding of what GES is and how the criteria that describe it may change over time. While data from the CPR will play a key role in this, net haul data as generated in this study will make an important contribution owing to the variety of physical and chemical parameters collected that will further our understanding of the environment the phytoplankton community inhabits.

## Supporting Information

S1 Table All data points for water temperature (temp), salinity (salin), density (sigma0), light attenuation (atten), turbidity and nutrients in the mixed surface layer and the average value for each environmental factor in the mixed surface layer. (XLSX)
